# A Genetic-Based Feature Selection Approach in the Identification of Left/Right Hand Motor Imagery for a Brain-Computer Interface

**DOI:** 10.3390/brainsci7010012

**Published:** 2017-01-23

**Authors:** Charles Yaacoub, Georges Mhanna, Sandy Rihana

**Affiliations:** Faculty of Engineering, Holy Spirit University of Kaslik (USEK), P.O. Box 446, Jounieh, Lebanon; georges.a.mhanna@net.usek.edu.lb (G.M.); sandyrihana@usek.edu.lb (S.R.)

**Keywords:** brain-computer interface, feature selection, genetic algorithm, hand motor imagery, neural networks

## Abstract

Electroencephalography is a non-invasive measure of the brain electrical activity generated by millions of neurons. Feature extraction in electroencephalography analysis is a core issue that may lead to accurate brain mental state classification. This paper presents a new feature selection method that improves left/right hand movement identification of a motor imagery brain-computer interface, based on genetic algorithms and artificial neural networks used as classifiers. Raw electroencephalography signals are first preprocessed using appropriate filtering. Feature extraction is carried out afterwards, based on spectral and temporal signal components, and thus a feature vector is constructed. As various features might be inaccurate and mislead the classifier, thus degrading the overall system performance, the proposed approach identifies a subset of features from a large feature space, such that the classifier error rate is reduced. Experimental results show that the proposed method is able to reduce the number of features to as low as 0.5% (i.e., the number of ignored features can reach 99.5%) while improving the accuracy, sensitivity, specificity, and precision of the classifier.

## 1. Introduction

The idea of a brain-computer interface (BCI) was originally introduced by Jacques Vidal in 1973 [1]. He demonstrated at the time that signals acquired from the brain can successfully inform about one’s intent. A BCI is a control or communication system in which the subject’s messages or commands do not rely on the brain’s usual output channels; in other words, the message is not carried by nerves and muscles. Moreover, in order to produce an action to carry a message or a command, neuromuscular activity is not needed [2].

Amyotrophic Lateral Sclerosis (ALS) and many other neurodegenerative diseases attack the nervous system, thus affecting the neural pathways between the brain and the different body organs. BCI is capable of bypassing the affected neuronal pathways by establishing communication between the human brain and an output device, which could be, for example, a robot, an artificial limb, or a computer [2]. A BCI can be applied in other applications as well (i.e., non-medical), such as computer and mobile gaming [3], and military affairs [4].

In order to accurately determine user intention, such as for example, spelling a word or performing a certain movement, features must be extracted from the acquired brain signals, and be processed and analyzed, to be later translated into commands. Signal processing techniques are thus crucial for the development of a BCI capable of achieving desirable results in various application fields.

The method of acquiring electrical signals from the brain is not unique. In fact, detecting brain activity can be invasive, partially invasive, or non-invasive. Actually, it all depends on the application and on the nature of the electrodes used to acquire the signals. Electroencephalography (EEG) is a popular non-invasive method of acquiring brain signals. The ease of use, portability, reduced cost, and high temporal resolution, among other characteristics, make the EEG the most widely used technique for acquiring brain signals [5]; it is extensively used to monitor and diagnose epilepsy, head trauma, brain tumors, sleep disorders, and other brain conditions.

Practical BCI systems require a minimal error rate to allow for real-time applications in both the medical and gaming fields. Choosing a suitable set of features might be a challenging task, since many features might be confusing to the signal classifier. In this paper, a feature selection method is proposed to determine the features that best differentiate between left and right hand motor imagery tasks based on the μ (i.e., 8–12.5 Hz) and β rhythms (i.e., the 13–30 Hz frequency band) of EEG signals, which occur during motor imagery and concentration, respectively [6].

An automated classification of left/right hand movement signals was presented in [7], where the Physionet database [8] was used and data was taken only from the C3, C4 and Cz channels, which are related to the left/right hand motor imagery (LRHMI). Filtering and automatic artifact removal were then carried out as part of the preprocessing stage using the EEGLAB toolbox [9]. Epoch extraction was performed taking in consideration event-related synchronization/de-synchronization (ERS/ERD) which are changes in the μ and β waves during movement or its imagination, and the movement-related cortical potentials (MRCP) which are a change in *δ* waves (i.e., 0.5–4 Hz frequency band) [7]. A rhythm isolation was carried out next using digital bandpass filters. The power, mean, energy, type (ERD/ERS, MRCP), and side (left or right) were taken as features. Neural networks and support vector machine (SVM) with an Anova–Kernel [7] were then used for classification.

Another approach was adopted in [10], based on discrete wavelet transform (DWT) coefficients and auto-regressive (AR) model parameters as features, and linear discriminant analysis (LDA) as a classifier. Other works with similar approaches include the usage of amplitude estimators as features [11], power spectral density and a Hamming window [12]. In a study by Cheng et al. [13], DWT coefficients were also used, and SVM outperformed LDA in classifying the signals.

Bou Assi et al. [14,15] applied the automatic K-means Independent Component Analysis (ICA) denoising method on multichannel EEG signals. They demonstrated a significant difference between relax and motor imagery using three features: the band power feature over C3, the coherence between C3 and C4 applied on level 4 detail coefficients of the Daubechies wavelet decomposition, and finally the phase locking value (PLV) between C3 and Cz after DWT. Using LDA as classifier, the average accuracy reached 88.10%, computed on 50 signals from different patients.

Other methods and BCI systems included a genetic algorithm (GA) for the selection of the features that best differentiate between the two classes, left/right hand motor imagery. A feature selection method was proposed in [16] that chooses spectral features, continuous wavelet transform, discrete wavelet transform, autoregressive model and μ-rhythm matched filter as methods for feature extraction. A feature set is therefore constructed and constitutes a chromosome for the genetic algorithm. Each bit of the chromosome represents a feature in the feature set. The fitness function for the GA is chosen as the kappa coefficient [16], a system evaluation criterion. The GA provides the best features and maximizes the kappa coefficient and therefore, the overall system performance.

In [17], the authors propose a similar approach, with an additional implementation of higher-order statistics in feature extraction, namely the variance, constructed as the power of the variant part of the signal, the kurtosis, which measures the extent of the signal distribution flatness, and the skewness, being the asymmetry of the signal distribution. The GA was run ten times and the most chosen features over all the runs were the ultimate optimal solution for the system. The error rate obtained was as low as 11%. In [18], each chromosome was encoded as a concatenation of the channel index, frequency, time index and the state of each signal, and a special fitness function was used. In [19], the GA was not used for feature selection, but rather for choosing the optimal parameters of the SVM classifier kernels.

In all previous studies, solutions are designed given a fixed set of features and pre-selected classifiers. Our paper proposes a genetic algorithm that optimizes feature selection for any given classifier, in general, regardless of the feature set. In other words, any feature could be included in the initial feature set, and any classifier can be used as well, without any change to the system architecture and functionality. The aim of the genetic algorithm is the reduction of the feature space while at the same time, optimizing the classifier output. The fitness function used in the GA aims at improving classifier performance by maximizing classification accuracy. The use of an artificial neural network (ANN) as a classifier along with a GA for feature selection not only allows outperformance of existing BCI systems for left/right hand movement identification, but also enormously reduces the feature vector length while, at the same time improving the classifier performance. As the GA is intended to be used offline (while training the system for a given target), the reduced feature vector decreases the computational burden at the classifier, thus reducing the overall online system (running without the GA) complexity. While other methods can also be used for dimensionality reduction such as principal component analysis (PCA), independent component analysis (ICA), and others, a main objective of this paper is to demonstrate and evaluate the usability, feasibility, and potential of genetic algorithms in the context of identifying left/right hand motor imagery for BCI systems. Furthermore, by using the GA, dimensionality reduction is not performed independently from the classifier in use, but suitable features are rather selected based on a feedback from the classifier, thus jointly optimizing feature selection and classifier performance.

The remainder of this paper is organized as follows. Section 2 presents the dataset, feature extraction and classification used in this study. In Section 3, the proposed genetic algorithm that reduces the feature vector while optimizing classifier’s performance is discussed. The experimental setup is then presented in Section 4 along with performance evaluation and analysis. Finally, conclusions are drawn in Section 5 with perspectives for future work extensions.

## 2. Materials and Methods

Preprocessing, feature extraction and classification, are three main stages in any BCI system. They can be performed in various ways and techniques, and each technique has its own flaws and benefits, which makes the design of a BCI system a challenging task, especially since EEG signals are non-stationary by nature. In the sequel, these stages will be presented within the context of our proposed approach.

### 2.1. Dataset and Preprocessing

**Figure 1:**
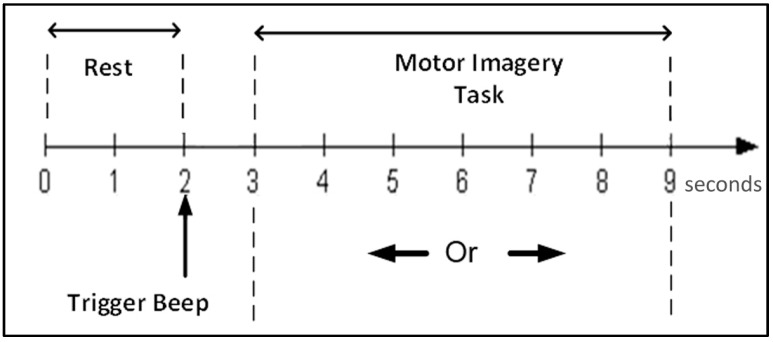
Graz dataset experience paradigm.

In our work, we rely on the Graz dataset III published for the 2003 BCI Competition [20]. Denote *t* a timing reference variable initialized as *t* = 0. Controlling a feedback bar using only the left/right hand imagery was the sole task in this experiment. First, the subject is at rest for two seconds. At *t* = 2 s, a beep announces the start of a single trial, and a visual stimulus appears as a cross for 1 s. At *t* = 3 s, a left or right arrow appears indicating the direction along which the subject has to move a bar. The subject performs the motor imagery task from *t* = 3 s to *t* = 9 s, at which the movement halts indicating the end of a single trial. The experience paradigm is depicted in Figure 1.

**Figure 2:**
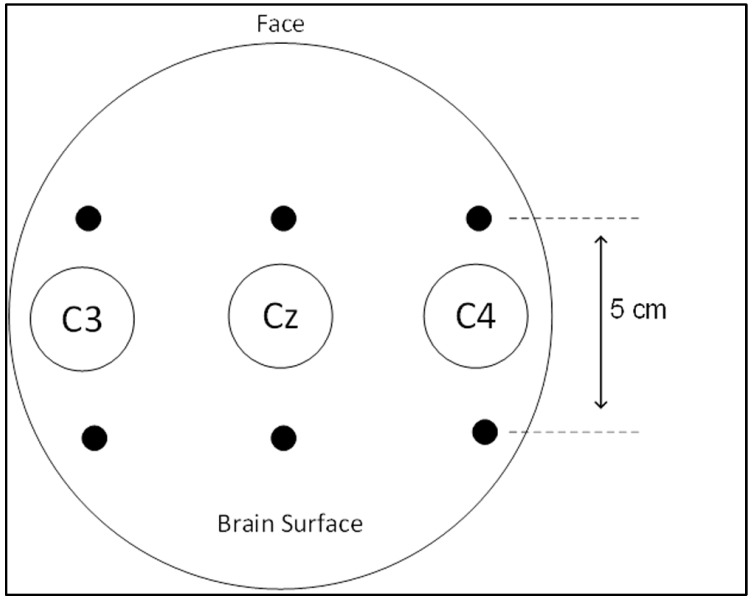
Electroencephalography (EEG) electrode placement.

Three brain channels were recorded over the C3, C4 and Cz electrodes, positioned as shown in Figure 2. C3 and C4 were included in the acquisition process since their respective signals are the most reliable when it comes to left and right motor imagery respectively [10]. Cz was only used as a reference. The dataset includes 140 nine-second trials, with a sampling frequency of 128 Hz, which results in 1152 samples per trial. A label vector is provided as well, indicating the class (left or right) of each trial.

A digital finite impulse response (FIR) elliptic bandpass filter is applied to each signal in the dataset, such that frequencies outside the interval (0.5 Hz, 30 Hz) are filtered out, thus eliminating muscle movement and power line interferences. In order to have zero-phase filtering, the FIR filter is first applied to the signal (i.e., forward filtering), then applied again after inverting the signal in time (i.e., backward filtering), and finally the signal is inverted back [5].

### 2.2. Feature Extraction

Signal properties have to be extracted before classifying the EEG signals according to their classes (i.e., left or right). For each trial, a sliding window of 3 s (i.e., 384 consecutive samples) is considered. Feature extraction within a 3-s window starts at time *t* = 2 s, i.e., at the moment when the subject starts thinking unconsciously of the direction it needs to move the cue. The window is moved sample by sample and features are extracted at every window position, until the end of the trial is reached, thus resulting in a total of 514 feature windows for each of the 140 trials.

The extracted features consist of autoregressive (AR) parameters as well as discrete wavelet transform (DWT) coefficient properties. An autoregressive model of order 6 is considered, as suggested in [21], which results in six features per channel, for each of the C3 and C4 channels. As for the DWT, the Daubechies order 5 mother wavelet (DB5) was considered based on [17]. Three levels of DWT are applied in order to reach the frequency band of interest: since motor imagery tasks differ primarily on the β and μ rhythms, the decomposed signals of interest are the detail coefficients of levels 2 and 3, respectively occupying the 16–32 Hz and 8–16 Hz frequency bands as shown in Table 1. From each detail signal in the selected bands, average amplitude, average band power and standard deviation of wavelet coefficients are computed as features. These features are chosen because of their diversity when it comes to what they represent; the first is a time-domain property of the signal window, the second is a frequency-domain property, while the third is of a statistical nature. As a result, a total of 24 features (cf. Table 2) are extracted in each time window, which makes a feature vector of 12,336 elements for each trial. Thus, a 140 by 12,336-feature matrix is obtained.

### 2.3. Classification

Once the features have been extracted and grouped into a feature vector, classification takes place, where each sample or trial are classified in one of the two classes: left or right hand motor imagery. In this work, artificial neural networks (ANN) [22] will be used as a classifier which, while being trained with samples whose classes are known, is capable of learning and adapting, to finally classify a new set of data whose classes are unknown.

Neural networks are a powerful technique to solve many real-world problems, based on the collaboration of multiple connected processing elements (neurons). Their performance is improved by their ability to learn from experience. They are capable of dealing with incomplete information. Inspired by the way biological nervous systems (e.g., the brain) process information, they can be used to detect patterns that are too complex to be perceived by humans or other computational techniques. To setup the ANN for classification, data should be divided into three main subsets:

- A training dataset to train the neural network
- A validation dataset to prevent overfitting and increase generalization
- A testing dataset to classify unseen data and evaluate the generalization

Classifier performance can be determined using several criteria:

- Sensitivity, which indicates the fraction of positives correctly classified as such, computed as:
  (1) $$\mathrm{Sensitivity}=\frac{TP}{TP+FN}$$
  where *P* stands for positive, *N* for negative, *T* for true, and *F* for false (e.g., *TP* is true positive, *FN* is false negative).
- Specificity, which indicates the fraction of negatives correctly classified as such, defined as:
  (2) $$\mathrm{Specificity}=\frac{TN}{TN+FP}$$
- Precision, which is the percentage of retrieved samples that are relevant, evaluated as:
  (3) $$\mathrm{Precision}=\frac{TP}{TP+FP}$$
- Accuracy, which indicates the fraction of correctly classified samples among all the samples, obtained by:
  (4) $$\mathrm{Accuracy}=\frac{TP+TN}{TP+TN+FP+FN}$$

To further validate the results, *K*-fold cross validation [23] will be used. It consists of:

- Randomly splitting the training set into *K* separate subsets that have approximately the same size and class proportions of the training set.
- Training the ANN using (*K*-2) subsets. One of the two remaining subsets will be used for validation and the other one for testing.
- Repeating the two previous operations *K* times, while a different subset is used for testing and Equation (1) through (4) are calculated at each repetition. The averages of sensitivity, specificity, precision, and/or accuracy are then computed.

## 3. Genetic Algorithm for Feature Selection

In this paper, we propose limiting the feature space when performing classification. This is aimed to reduce system complexity and eliminate features that could be misleading to the classifier. A genetic algorithm will be used for this purpose.

**Figure 3:**
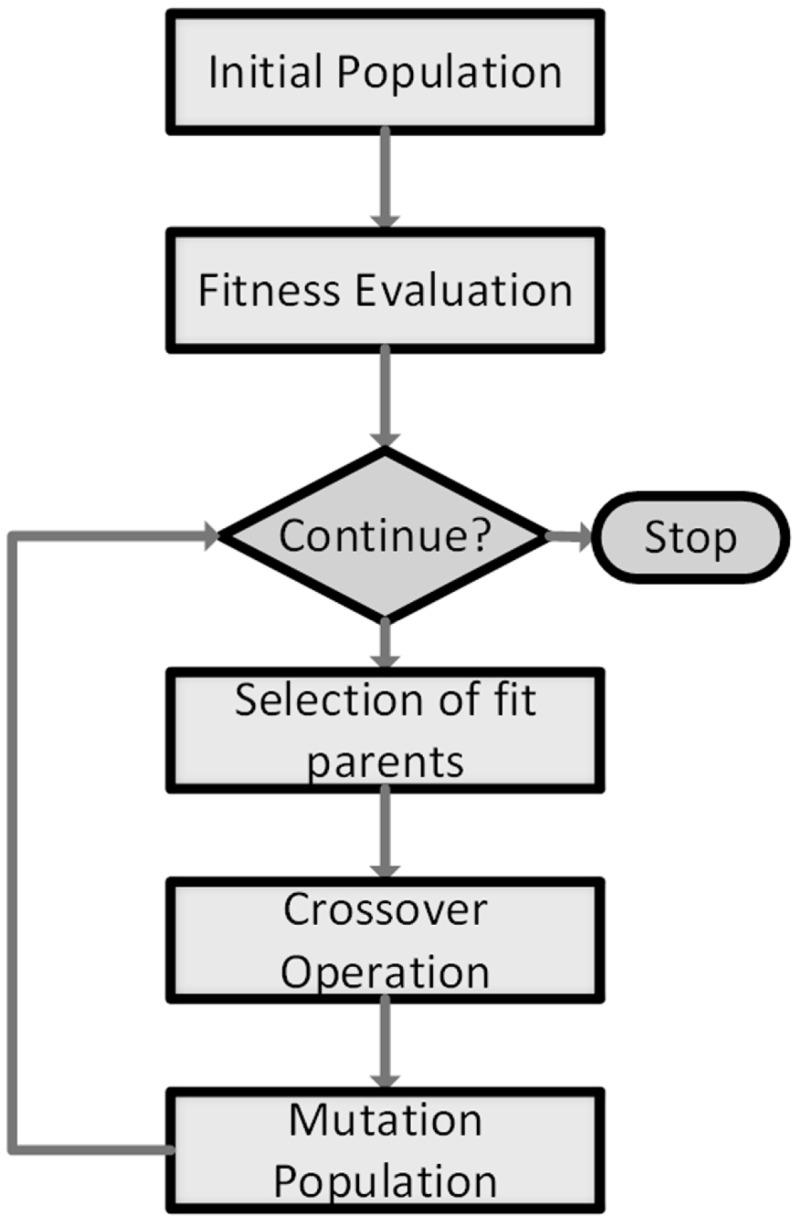
Flowchart diagram of a basic genetic algorithm (GA).

Genetic algorithms are based on the principles of evolution and natural genetics [24], and are mainly used to numerically solve optimization problems. In a GA, a possible solution vector is referred as a chromosome, which consists of a set of genes. A population is a set of chromosomes in the solution space. Generally, a GA operates as illustrated in Figure 3, explained hereafter.

First, an initial population of *N* chromosomes is defined, each of length *L*. A fitness function is then evaluated for each chromosome in the population. Chromosomes are selected to be parents and recombine, thus reproducing new offspring. Naturally, parents should be fit enough to reproduce. Therefore, a probability of selection (i.e., probability for a particular chromosome to be selected as a parent) should depend on the fitness function. A method for assigning selection probabilities is the roulette wheel method [25]; if *x_i_* is the *i*-th chromosome in the population and *f* (*x_i_*) its fitness, then the selection probability would be:

(5) $$P_{s}=\frac{f(x_{i})}{\sum_{i=1}^{N}f(x_{i})}$$

In the crossover operation, selected parents are merged together and new children are formed. Mutation consists of randomly altering genes inside chromosomes, with a very low probability; this allows the GA to escape converging towards local optima. A new population is thus formed replacing the previous one. Selection, crossover and mutation are iteratively applied until some stopping criterion is met or a predefined maximum number of iterations is reached [26]. A variation of the basic GA is introduced to improve its performance, by applying elitism, which consists of preserving the fittest chromosome in a population for the next generation. This mitigates the risk of losing this chromosome by crossover or mutation and allows for a faster convergence towards the optimal solution.

**Figure 4:**
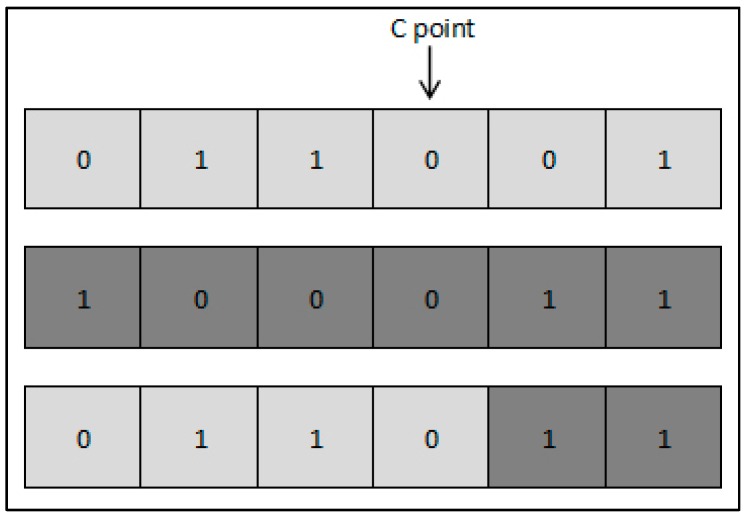
Crossover example using chromosomes of length *L* = 6.

In this paper, by using the GA we aim at selecting the feature set that allows for the best classification of left/right motor imagery given EEG signals. Therefore, problem formulation to the context of the GA is required. As earlier mentioned, our feature vector consists of 12,336 different features; thus, a chromosome is represented with a binary vector of length *L* = 12,336, where each index represents one feature. A binary “1” indicates that the feature at the same index will be used for classification, and “0” indicates that the feature will be ignored. The first population is initialized by randomly filling the feature vectors with binary data, while the probability of a given binary symbol and the population size are set as input parameters. We consider the accuracy of the classifier as the fitness function that the GA will seek to maximize. Therefore, for each chromosome, a neural network is created, trained and tested, to finally obtain the classifier’s accuracy. Parents are selected with probability *P_s_* as defined in (5), and crossover operation is then applied. For each couple of selected parents, the crossover point (C point) is selected as a random position and new offspring are then created. Crossover operation is illustrated in Figure 4; a child is created such that all genes before the C point are copied from the first parent, and the remaining genes following the C point are copied from the second parent. Mutation is then applied to enlarge the search space and mitigate the risk of converging towards local optima; it consists of randomly flipping a value within a chromosome, and occurs with a probability *P_m_* predefined as an input parameter.

**Figure 5:**
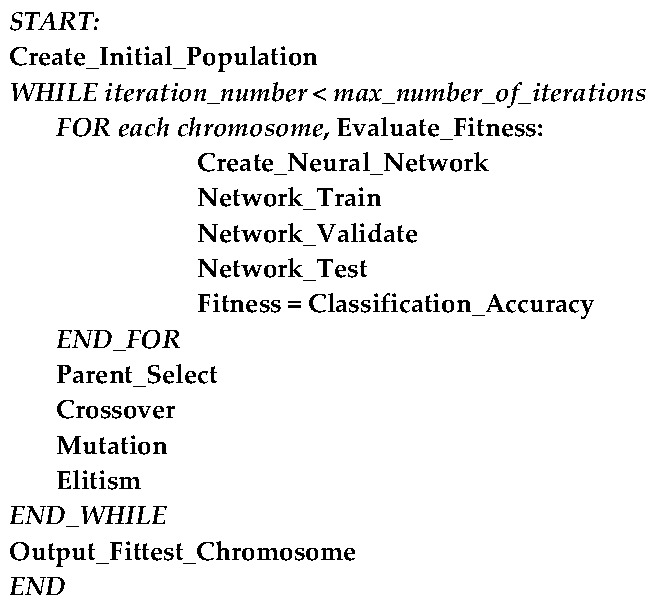
Pseudocode of the proposed GA-based feature selection algorithm. GA: genetic algorithm.

After the last iteration, the GA outputs a chromosome that indicates the best solution, i.e., the feature subset offering the best classification accuracy. A pseudocode summarizing our GA-based feature selection algorithm is shown in Figure 5.

## 4. Experimental Results and Discussion

This section evaluates the performance of the proposed approach for feature selection, by first analyzing the ANN as a classifier that relies on all available features (i.e., 12,336) for each signal, then showing the advantages of including the GA in the feature selection process. Despite the additional complexity of the GA, the algorithm is intended to run offline in order to identify a small feature subset that can be later used for real-time classification, thus reducing the overall complexity of the intended final BCI system. As for the ANN, the number of neurons in the hidden layer has been varied from 5 to 100. It was observed that the system’s accuracy does not monotonously improve with the number of neurons, but rather slightly fluctuates around a constant value, while larger values induce additional complexity and simulation time. Therefore, any value could be used without loss of generality of the results presented hereafter.

### 4.1. ANN-Based Classification without GA-Based Feature Selection

The first experiment consists of classifying the signals without feature selection, i.e., considering all available features for each signal. The number of neurons in the hidden layer is 7, and the EEG signal database is divided such that 70% of the samples were used for training, 15% for validation, and 15% for testing. In other words, the 140 available samples are randomly distributed such that 98 samples are used for training, 21 for validation and 21 for testing. Table 3 shows the confusion matrices obtained at each of the training, validation, and testing phases, as well as the matrix obtained considering the results of all phases. Even though GA-based feature selection has not been applied, our designed system outperforms different other solutions available in the literature as shown in Table 4 where, for fair comparison, only systems using the same signal database are considered. These systems mainly differ by their feature vector (i.e., features used for classification) and classifier. It will be later shown that, by including the proposed approach for feature selection, performance will be further improved.

To further validate our results, *K*-fold cross validation has been applied with *K* = 5 and *K* = 7. In the former case we have 28 samples per fold (i.e., 84 samples for training, 28 for validation, and 28 for testing), and with the latter we obtain 20 samples per fold. Using higher values of *K* would not be suitable in our case, since the number of samples per fold would not be sufficient to accurately evaluate the system’s performance. Validation results in terms of accuracy are summarized in Table 5. It can be noticed that the average testing accuracy is 91.43% for *K* = 5, and 92.86% for *K* = 7.

### 4.2. Accuracy Maximization with GA-Based Feature Selection

All the trials conducted this far are related to the classification process itself. The results presented this point onwards include the GA-based feature selection.

In the following experiment, we use 50% of samples for training, 30% for validation, and 20% for testing. As for the GA parameters, 20 iterations are performed, with a population size of 50 chromosomes and mutation probability *P_m_* = 5% (based on [17] and [28]). Chromosomes of the initial population are initialized with equally likely symbols (zeros and ones). Note that in this experiment, no cross-validation is performed.

**Figure 6:**
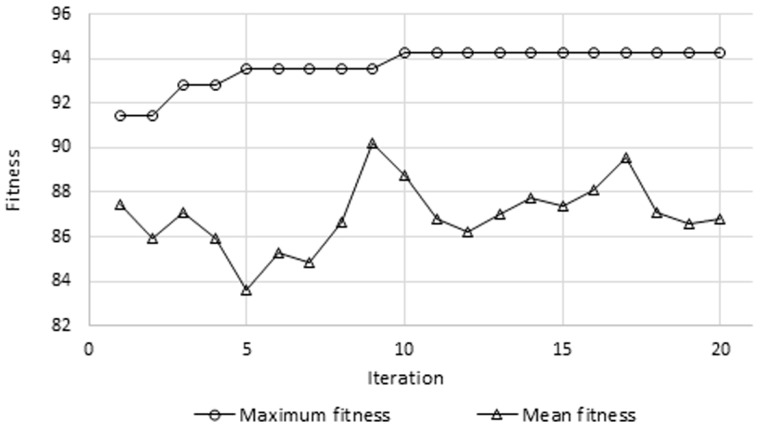
Mean and maximum fitness values at different iterations of the genetic algorithm.

**Figure 7:**
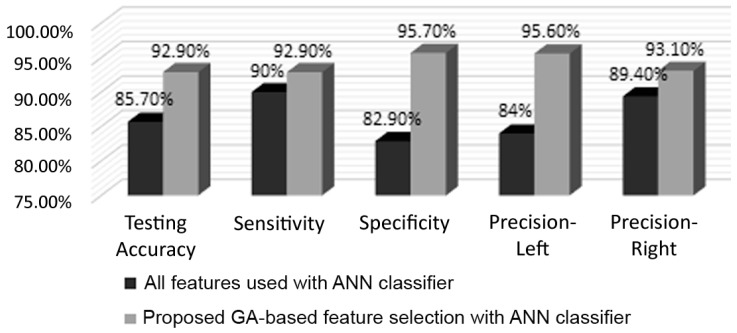
Comparison of ANN classifier performance with and without GA-based feature selection. ANN: artificial neural network. GA: genetic algorithm.

Figure 6 presents the variations of the mean and maximum fitness values after each iteration of the GA. It can be observed that highest accuracy was obtained at the tenth iteration and remained constant afterwards, whereas the mean value kept fluctuating. This shows that during the last 10 iterations, through crossover, mutation, and elitism, the GA was not able to generate a new chromosome (i.e., feature set) that allows for a higher classification accuracy. On the other hand, Figure 7 depicts a comparison highlighting the efficacy of the GA-based selection, based on the multiple evaluation criteria previously explained. It can be clearly observed that considerable improvement was incurred due to the GA. For example, the testing accuracy increased from 85.7% to 92.9%, sensitivity increased from 90% to 92.9%, and a gain of 12.8% was obtained in terms of specificity. As for left and right precisions, they improved by 11.6% and 3.7%, respectively. The GA not only improved performance, but also reduced the number of features required for classification by roughly 53%, as the length of the feature vector was reduced from 12,336 to 5817.

The simulations were run on a computer with Windows 10, 8GB of memory, and a 4-core Intel Core I7 with hyperthreading capability. Under this configuration, each iteration of the GA requires between 40 and 60 s to execute, with an average of 45 s/iteration. While the execution time could be considered relatively high making its real-time application impossible in a BCI system, the GA suggested in this study is intended to run offline in order to determine a small subset of features to be later used in the real-time BCI application. It is also important to mention that the GA is easily parallelizable; therefore, with code optimization and parallel processing (e.g., using a graphics processing unit—GPU), the time required for the GA to execute can be reduced to a large extent.

On the other hand, though a number of 5817 features would still be large, the GA can be controlled so as not to exceed a predetermined number of features, depending on timing and complexity constraints that can be incurred due to physical considerations of the BCI. For example, any chromosome containing a number of 1’s greater than a pre-defined threshold can be assigned a fitness value of 0, such that it will be eliminated in the evolutionary process of the GA. Another approach consists of initializing the GA with a population where the selected features are scarce (relative to the initial feature space), as in the experimental setup of Section 4.3.

**Figure 8:**
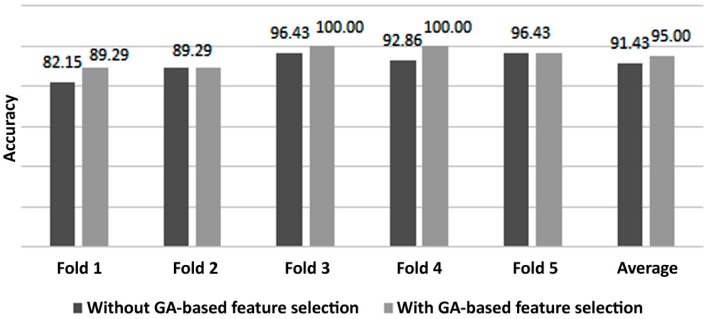
A 5-fold cross-validation of classifier accuracy with and without GA-based feature selection. GA: genetic algorithm.

The next experiment consists of modifying the fitness function of the GA, such that a 5-fold cross validation is performed and the fitness function is defined as the average value of the testing accuracy. The same initial conditions apply. The observed fitness value on the first iteration is close to 91% and evolves towards an optimal solution across generations. The GA converges after 15 generations with a maximum fitness of 95%. Figure 8 depicts a comparison between the classification results of a 5-fold cross validation performed with and without the proposed feature selection. It can be noticed from Figure 8 that the GA was able to improve the 5-fold cross validation results as well. It is also noted that the same folds were applied in both cases (with and without feature selection) in order not to change the initial conditions and thus, to fairly compare the results. It is important to mention that for some folds, the same fitness value is obtained whether feature selection is applied or not, which may falsely indicate that the GA failed to improve the system’s performance. In fact, for these folds (e.g., fold 2 and fold 5 in Figure 8), the same accuracy is obtained with roughly half the features, selected by the GA, compared to the case where the GA was not applied. In other words, the ANN classifier can yield the same accuracy while significantly reducing its input feature vector, which results in reducing the complexity of the final BCI system.

On the other hand, a *t*-test [29] was applied to analyze the statistical significance of the differences in accuracies after verifying the normality of the data using a Kolmogorov–Smirnov test [30], and statistical significance was observed at a significance level of 9%. It is important to mention that this significance level is obtained given the limited number of samples available in the database (i.e., 140 samples of which a subset is used for testing); a smaller significance level could be obtained if a larger database was used.

### 4.3. Initializing the GA with Limited Number of Features per Chromosome

Up to this point, the probability for a gene to have a value of “1” or “0” in a chromosome, during the initialization phase of the GA, was 50%. In other words, each feature had equal chances of being included or not in the feature vector. In the following experiment, the probability of initially including a feature in a given feature vector is reduced to 0.25% (i.e., out of the 12,336 features, roughly 30 features are randomly selected), the mutation probability was reduced to 0.1% to decrease the chances of bit flipping, and the number of neurons in the hidden layer of the ANN is 5. All other parameters remain unchanged.

After the initial conditions have been set, feature selection is carried out. The fitness attains a maximum value of 92.1% from the eighth generation, and the fittest chromosome finally obtained contains 61 selected features, which are then used for classification. One would expect the fittest chromosome to have the same number of selected features as in the previous experiment of Section 4.2, since the fitness function that the GA aims at optimizing, as well as the initial feature set, are the same in both scenarios. The obtained results can be explained by the fact that the GA could have converged towards a local optimum, and would require a larger number of iterations and/or different parameter values (crossover probability, mutation probability, population size, …) in order to converge towards a same (optimal) result every time.

**Figure 9:**
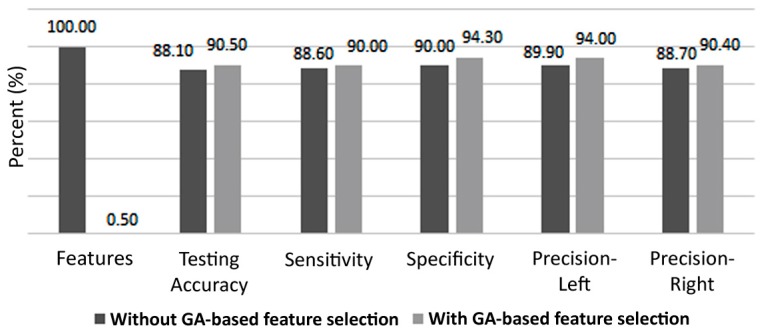
Classification results (12,336 vs. 61 features).

Figure 9 shows classifier performance with and without feature selection. Even though only slight improvement is observed, it is crucial to mention that the number of features needed for classification, in this experiment, has been reduced by 99.5%, which allows for a tremendous reduction in classifier complexity.

**Figure 10:**
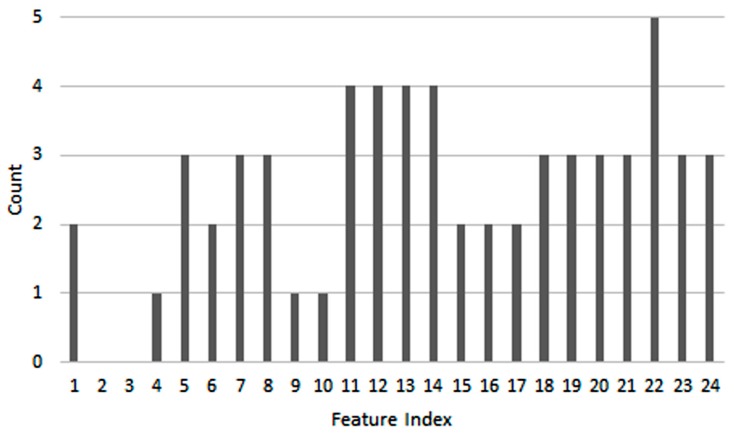
Feature histogram in the fittest chromosome.

By analyzing the feature histogram in the fittest chromosome (Figure 10), it can be observed that out of the 61 selected features, 24 are AR parameters whereas 37 are DWT coefficients, which indicates that the DWT has a stronger contribution to the classification results compared to the AR model. On the other hand, based on Figure 10 and referring to Table 2, it can be noticed that 29 of the features are obtained from the C3 channel, whereas 32 are from C4; this shows that the number of features from both channels is roughly equal.

**Figure 11:**
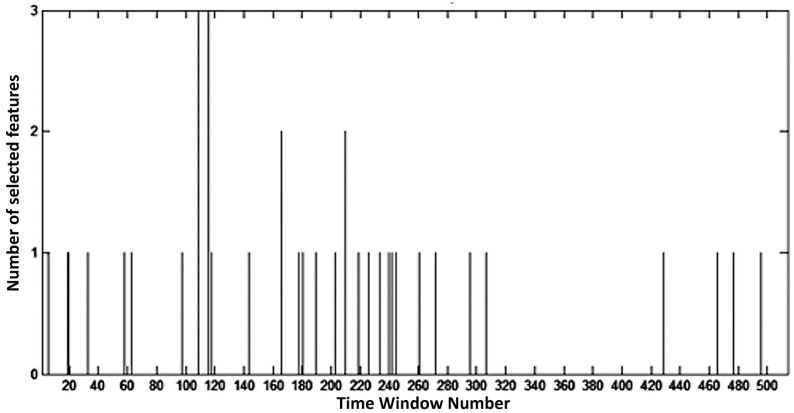
Number of selected features per time window.

Figure 11 shows the number of features selected by the GA in each of the 514 time windows (of 3 s each). It can be observed that no feature was selected between the windows 310 and 420, whereas most features are extracted during the first 310 time windows, or in other words, from windows starting between the trigger beep (Figure 1) and 1.4 s after the beginning of hand motor imagery. Therefore, according to the GA, this time interval is the most important for classification performance.

## 5. Conclusions

In this paper, a feature selection approach for the identification of left/right hand motor imagery was presented, based on genetic algorithms, in the context of a brain-computer interface. The proposed approach relies on different features that were used in previous studies for the same purpose. However, as the number of features increases, the high computational load for the identification process makes the system unsuitable for real-time BCI applications. Furthermore, a large number of features would mislead the classifier and could result in reduced accuracy. Additionally, a different classifier would perform differently with different feature sets. In our study, the proposed approach could be used with any classifier in order to identify a subset of features that would lead to best performance. Practically, we suggested the use of ANN for classification due to their superior performance. Our results show that our proposed genetic algorithm is able to reduce the required number of features while at the same time improving the overall classifier’s performance. The average accuracy with the GA enabled increased by 3.6% with roughly half the features, compared to the case where all features were used. Even with only 0.5% of the features (i.e., a reduction of 99.5% of the feature vector size due to the proposed GA-based selection), classification accuracy increased by 1.7%. Since the GA is only used offline, during the system training phase, the additional complexity incurred by the GA does not affect the real-time performance of the BCI, which would rather have a faster response time due to the reduced number of required features.

As for future perspectives, we will be studying the effect of the different system parameters (GA parameters, ANN parameters, etc.) on the overall performance, as well as the utility of including more features in the initial feature space, with the aim of optimizing the parameter set (i.e., parameters resulting in optimal performance). Following this optimization, it is of utmost importance to study the system performance when applied on signals acquired in real-time, for the development of a prototype of our desired BCI.

## Figures and Tables

**Table 1:** Discrete wavelet transform (DWT) decomposition (*F_s_* = 128 Hz, DB5, three decomposition levels). DB5: Daubechies order 5 mother wavelet.

Level	Frequency Band (Hz)	Decomposed Signal
L1	32–64	Detail Coefficients
L2	16–32	Detail Coefficients
L3	8–16	Detail Coefficients
L3	0–8	Approximation Coefficients

**Table 2:** Feature vector structure for a single time window. AR: auto-regressive.

Index	Feature	Channel
1–6	AR parameters	C3
7–12	AR parameters	C4
13	Mean of Amplitudes of L2 Coefficients	C3
14	Mean of Amplitudes of L3 Coefficients	C3
15	Mean of Amplitudes of L2 Coefficients	C4
16	Mean of Amplitudes of L3 Coefficients	C4
17	Standard Deviation of L2 coefficients	C3
18	Standard Deviation of L3 coefficients	C3
19	Standard Deviation of L2 coefficients	C4
20	Standard Deviation of L3 coefficients	C4
21	Average Power of L2 coefficients	C3
22	Average Power of L3 coefficients	C3
23	Average Power of L2 coefficients	C4
24	Average Power of L3 coefficients	C4

**Table 3:** Confusion matrices of artificial neural networks (ANN) classifier without GA-based feature selection. GA: genetic algorithm.

			Predicted		
			Left	Right	
**Actual**	**Training Samples**	Left	45	3	Sensitivity 93.8%
		Right	5	45	Specificity 90%
			Precision 90%	Precision 93.8%	Accuracy 91.8%
	**Validation Samples**	Left	9	0	Sensitivity 100%
		Right	1	11	Specificity 91.7%
			Precision 90%	Precision 100%	Accuracy 95.2%
	**Testing Samples**	Left	13	0	Sensitivity 100%
		Right	2	6	Specificity 75%
			Precision 86.7%	Precision 100%	Accuracy 90.5%
	**All Samples**	Left	67	3	Sensitivity 95.7%
		Right	8	62	Specificity 88.6%
			Precision 89.3%	Precision 95.4%	Accuracy 92.1%

**Table 4:** Comparison of testing accuracy with different systems. BCI: brain-computer interface. GA: genetic algorithm.

Classification System	Accuracy
Proposed (before GA-based feature selection)	90.5%
Method of [10]	90%
Method of [27]	89.29%
Graz 2003 BCI Competition [20], first place	89.29%
Graz 2003 BCI Competition [20], second place	84.29%
Graz 2003 BCI Competition [20], third place	82.86%

**Table 5:** Artificial Neural Network (ANN) classifier accuracy with *K*-fold cross-validation.

*K*	Fold Number	Training Accuracy (%)	Testing Accuracy (%)	Total Accuracy (%)
5	1	96.43	82.15	92.15
	2	91.67	89.29	92.15
	3	89.29	96.43	92.15
	4	90.48	92.86	92.15
	5	96.43	96.43	93.58
	Average:	92.86	91.43	92.44
7	1	94.00	95.00	91.43
	2	95.00	75.00	92.86
	3	91.00	100.00	92.86
	4	91.00	95.00	92.86
	5	92.00	100.00	92.86
	6	93.00	90.00	92.86
	7	94.00	95.00	95.00
	Average:	92.86	92.86	92.96
